# Real and perceived barriers to effective use of cataract surgical supplies

**DOI:** 10.1016/j.joclim.2026.100664

**Published:** 2026-04-16

**Authors:** Caitlin Davie, Lindsay K. Cloud, Scott Burris, Shaina Shiwdin, Brooke Sherry, Christina R. Prescott, Joel S. Schuman, David Palmer, David Chang, Daniel Parra, Cordelia Kwon, Margaret A. Tharp, Cassandra L. Thiel

**Affiliations:** a Legal Program Manager, Center Public Health Law Research, Temple University Beasley School of Law, Philadelphia, PA 19122, USA; b Deputy Director of the Policy Surveillance Program, Center Public Health Law Research, Temple University Beasley School of Law, Philadelphia, PA 19122, USA; c Barnett College of Public Health, Temple University, Philadelphia, PA 19122, USA; d Center for Public Health Law Research, Temple University Beasley School of Law, Philadelphia, PA 19122, USA; e Health Department of Population Health, NYU Langone Health, New York, NY 10016, USA; f University of Texas Health Science Center, Houston, TX 77030, USA; g Vice Chair for Ophthalmology Education, Director of Cornea Service, Department of Ophthalmology, NYU Langone Health, New York NY, 10016, USA; h Kenneth L. Roper, MD Endowed Chair; Vice Chair for Research Innovation; Co-Director Advanced Center for Ophthalmic Research in Neuroimaging (ACORN); Vickie and Jack Farber Vision Research Center; Co-Director Glaucoma Service, Wills Eye Hospital, Philadelphia, PA, 19107, USA; i Professor, Department of Ophthalmology, Sidney Kimmel Medical College at Thomas Jefferson University, Philadelphia, PA, 19107, USA; j Professor of Biomedical Engineering, Drexel University School of Biomedical Engineering, Science and Health Systems, Philadelphia, PA, 19104, USA; k Clinical Associate Professor, Department of Ophthalmology, Northwestern University Feinberg School of Medicine, Chicago, IL 60611, USA; l Clinical Professor of Ophthalmology, UCSF, Chair, ASCRS Foundation, San Francisco, CA, 94158, USA; m The Warren Alpert Medical School of Brown University, Providence, RI, 02903, USA; n PhD Candidate Health Policy, Harvard University, Cambridge, MA, 02138, USA; o Indiana University School of Medicine, Indianapolis, IN, 46202, USA; p Assistant Professor Departments of Population Health, Ophthalmology NYU Langone Health, 227 E 30th St #636, NY, NY 10016, USA; q President and CEO, Clinically Sustainable Consulting, Madison, WI, 53562, USA

**Keywords:** Cataract, Waste, Malpractice, Policy, Infection, Regulation

## Abstract

**Introduction:**

Cataract surgery, among the most common procedures worldwide, significantly contributes to climate change through both solid waste and air pollution, highlighting the urgent need for targeted interventions. Implementation faces hurdles including legal constraints and concerns about infection risks and malpractice.

**Methods:**

This article examines federal and state regulatory structures around two key waste reduction strategies: reduction of multidose medications and distribution of partly-used operating room medication to patients post-surgery.

**Results:**

We find little-to-no legitimate regulatory barriers to either waste reduction practice. Dispensing and redistributing partly-used medication has been codified in law in some states. Evidence-based reports indicate minimal risks for infection with proper handling. From 1998 until August 2024, according to the Ophthalmic Mutual Insurance Company, there were no reported endophthalmitis lawsuits from using multidose eye drops perioperatively. Malpractice fears also appear to be generally unfounded considering guidelines by various professional organizations identifying these practices as acceptable standards of care and lack of related lawsuits.

**Conclusion:**

In order to implement evidence-based sustainability interventions, more education and training is needed to ensure clinicians and staff are aware of real and perceived barriers.

## 1 Introduction

Cataract surgery provides significant benefits to patients and society and is one of the United States’ (US’) most common procedures [1,2]. Medical waste leads to environmental damages, through generation of solid waste and greenhouse gases [3,4], and financial consequences, with annual costs estimated at $760-$935 billion in the US [5]. Implementing waste reduction interventions could save an estimated $12.8-$28.6 billion annually [6]. Drug waste is a key area for improvement in US ophthalmic practice [3,7]. Common interventions include multidosing topical medications (such as antibiotics, mydriatics, analgesics, etc.) and distributing partly-used operating room medications to patients post-operatively. Sending patients home with medication has minimal risks but is not conducted universally [8,9]. While multidosing carries a perceived risk of infection, multiple studies confirm that with application of accepted guidelines and proper training, multidosing is safe and does not increase the risk of endophthalmitis [[10], [11], [12]].

Despite the benefits, these sustainability interventions are not broadly adopted in US ophthalmic care [8]. This paper explores real and perceived regulatory barriers to implementing these practices. Real barriers include laws, regulations, and facility policies that prevent a surgeon or institution from applying these waste reduction practices. Perceived barriers may be based on fears of infection, malpractice, or a misunderstanding of the law.

## 2 Materials and methods

A narrative review of the legal and regulatory landscape governing waste reduction and environmental sustainability strategies in cataract surgery was conducted using the online legal research database Westlaw (Thomson Reuters, Eagan Minnesota, USA); legislative, regulatory, or policy records from federal and state websites; and available guidance from professional organizations such as The American Medical Association (AMA). Search phrases were constructed using combinations the following: reuse, single-use, multiuse, multidose, discharge, single-patient use, cataract surgery, medications, eye drops, post-operative. The research has been summarized into four categories (1) federal law, (2) state law, (3) professional medical organizations, and (4) hospital and Ambulatory Surgical Center (ASC) policies.

Additionally, a convenience sample of three experienced US-based cataract surgeons were interviewed (Table 1) to gain insight into current practices and decision making.

**Table 1:** Interview questions from informal survey.

(1) What are the rules that decide whether you or your colleagues reuse multi-dose eye drops and other drugs in the ASC/hospital?
(2) Where do these rules come from? (Please choose all that apply: State laws or regulations; Federal laws or regulations; Joint Commission; The hospital/ASC; The label of the medication; Malpractice insurance; Other)
(3) Do you have the authority to determine whether to reuse medications? (3a) If you do have the authority to decide to reuse and choose not to reuse, why? (i.e., what rules or concerns prevent you from re-using medication? Infection control, financial, attempting to avoid malpractice, etc.)
(4) We have noticed that eye drops in offices (ex. dilating drops) are often reused and shared between patients. Why do you think that is common in office situations but not during surgery? (4a) Is it because there is less bureaucracy? (4b) Is there less risk of infection? (4c) Other reasons?
(5) Are there other wasteful practices during surgery? (5a) Why are these practices being implemented?
(6) Are there areas you think we should cover/are missing?

## 3 Results

### 3.1 Interview responses

Interviewed surgeons stated that ability to reuse medications was generally determined at the state level, often through a state’s pharmacy practice act. State laws are influenced by the Center for Disease Control and Prevention (CDC) and Joint Commission (JC), with The Food and Drug Administration (FDA) also playing a role in labeling requirements. Surgeons also noted that facilities themselves may prohibit multidosing due to fears of infection risks or malpractice.

Surgeon interviews also revealed administrative concerns around billing and reimbursements for multidosing. Electronic medical record systems may not be able to bill patients per eye drop, or it may be too time-consuming to track. Without tracking appropriately, some facilities could lose federally subsidized medication discounts (340B Drug Pricing Program) for “under accumulation” of the eye drop bottles.

The interviewees also shed light on misunderstandings interfering with sustainability initiatives. One respondent noted a misconception stemming from a flawed 2015 Centers for Medicare & Medicaid Services (CMS) checklist, which incorrectly applied rules for injectables (single use and a 28-day expiration date) to eyedrops. Although CMS formally corrected this in a 2022 memorandum, the initial error's impact persists in practice [12,13].

### 3.2 Legal review

#### 3.2.1 Federal law

**Fig. 1:**
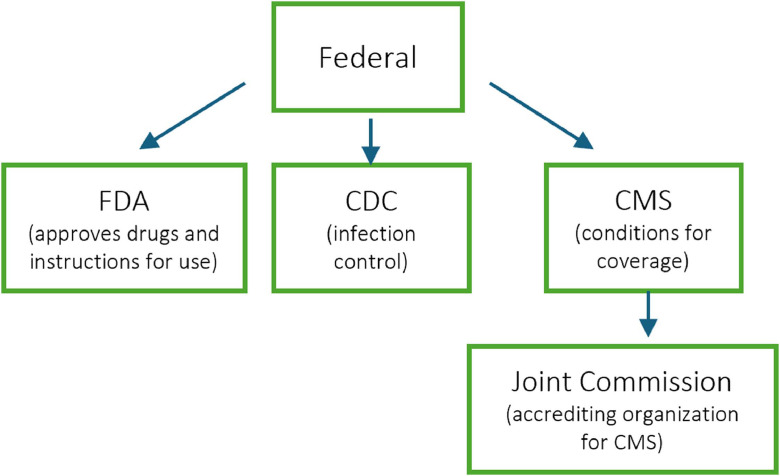
Map of relevant federal-level agencies impacting policies related to supply utilization in ophthalmology.

Federal regulation is influenced by multiple agencies (Fig. 1), but none explicitly prohibit waste reduction practices. The FDA regulates drug labeling. If a drug is not labeled as single-dose, it is not legally restricted to single-use [14,15]. Deviations from labels, such as off-label use, are generally not prima facie evidence of malpractice in all states, though labels can be used to determine the standard of care in malpractice trials in some states [16,17]. The Centers for Medicare & Medicaid Services (CMS) Conditions for Coverage (CfC) that health organizations must meet to participate in Medicare/Medicaid do not prohibit the use of multi-dose eye drop bottles for multiple patients [18,19]. CMS permits this practice if the solution is labeled multiuse and standard infectious precautions are followed [19].

The Joint Commission (JC) is one of several accrediting organizations for CMS and is an independent, not-for-profit organization, and in that role the JC establishes significant precedent [20,21]. Following a hospital appeal in 2012, the JC reversed its initial finding against multidosing eye drops, allowing the practice to resume so long as safe handling and administration guidelines from the American Academy of Ophthalmology were followed [10].

#### 3.2.2 State law

We found that no state laws prohibiting the multidosing of ophthalmic drops administered intraoperatively or the practice of dispensing leftover intraoperative topical medications for post-operative care. Laws addressing the reuse of medication primarily focus on pharmacy requirements. For example, Minnesota law Minn. R. 6800.2700 authorizes hospitals with pharmacies to return to the pharmacy for reuse drugs that were dispensed for inpatient use, so long as they have not left the span of control of the pharmacy, defined in Minn. R. 6800.7400 as the areas of the hospital where drugs are stored. In South Dakota, S.D. Admin. R. 20:51:13:02 permits hospitals with pharmacies to reuse drugs that were dispensed for inpatient use if the integrity of the drug and its packaging is maintained.

Importantly, some states have passed supportive laws explicitly authorizing or requiring waste reduction practices (Table 2). In Missouri, Mo. Code Regs. tit. 19, § 30–20.100, allows medication in multidose containers—such as ophthalmic drops—to be discharged with hospital patients when ordered by a qualified practitioner. The Tennessee Topical Medical Waste Reduction Act of 2023 states that if a medication used for a surgical procedure is ordered least twenty-four in advance, then the unused portions may be offered to the patient for continuing treatment. Illinois state law, 225 Ill. Comp. Stat. 85/15.10 requires that any unused portion of a facility-provided medication ordered at least 24 h in advance must be offered to the patient upon discharge.

**Table 2:** Summary of state laws explicitly authorizing patients to be send home with the unused medication from the patient’s surgery.

Law	Locations	Required	Permitted
Arizona			
Ariz. Rev. Stat. § 32–1934	Remote hospital-site pharmacy	X	
Arkansas			
Ark. Admin. Code 007.05.10–12	Critical Access Hospitals		x
Ark. Admin. Code 007.05.17–12	Hospitals		x
Missouri			
Mo. Code Regs. tit. 19, § 30–20.100	Hospitals		x
Illinois			
210 Ill. Comp. Stat. 85/6.30	Facility-provided*	X	
210 Ill. Comp. Stat. Ann. 5/7d	Ambulatory Surgical Treatment Center	X	
225 Ill. Comp. Stat. 85/15.10	Facility-provided*	X	
110 Ill. Comp. Stat. 330/8e	Facility-provided*	X	
Ill. Admin. Code tit. 77, § 250.240	Hospital	X	
Nebraska			
Neb. Rev. Stat. § 71–475	Hospital; Ambulatory surgical center; Health care practitioner facility	X	
South Carolina			
S.C. Code Regs. 61–16 § 1207	Hospitals		x
Tennessee			
Tenn. Code § 68–11–2204	Hospital operating room; hospital emergency room department		x
Utah			
Utah Code § 58–17b-602	Hospital pharmacy		x
Utah Admin. Code r. R432–500–19	Freestanding Ambulatory Surgical Center		x

⁎ Not defined further.

#### 3.2.3 Professional society consensus

**Fig. 2:**
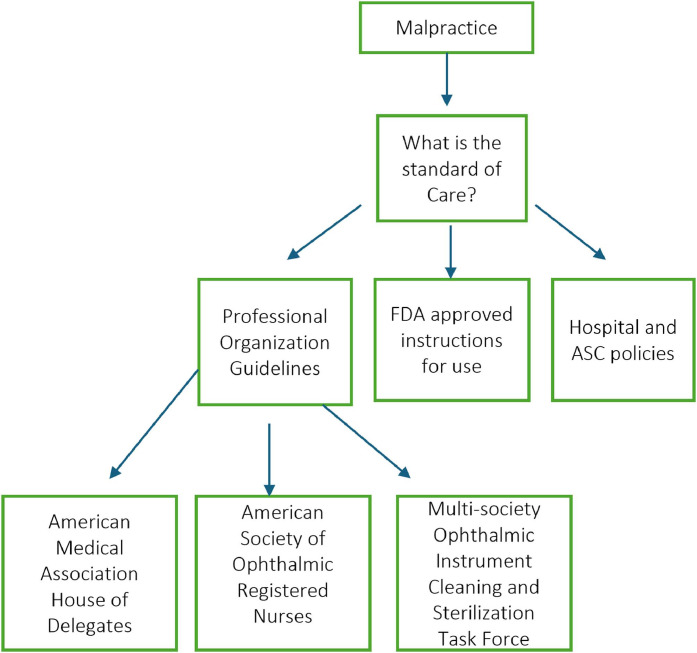
Map of policies, guidelines, and their supporting organizations that help establish standard of care in medical malpractice cases for ophthalmology.

Professional medical organizations consistently support these waste reduction practices, which helps establish the standard of care in potential malpractice cases (Fig. 2).

The American Society of Ophthalmic Registered Nurses (ASORN) publishes guidelines confirming that using multidose medications on multiple patients is allowed and safe if best practices, such as proper hand washing and strict no-patient-touch administration protocols, are followed [11,18,21,22]. This consensus has been confirmed by other organizations, including the Accreditation Association for Ambulatory Health Care (AAAHC) and QUADA (formerly American Association for Accreditation of Ambulatory Surgery Facilities- AAAASF) [21].

EyeSustain (previously the Ophthalmic Instrument Cleaning and Sterilization (OICS) Task Force) is an international multi-society organization, supported by the American Society of Cataract and Refractive Surgery (ASCRS), the American Academy of Ophthalmology (AAO), American Glaucoma Society (AGS), and the Outpatient Ophthalmic Surgery Society (OOSS), that actively advocates for waste reduction [22,23]. A survey of their members found that 93 % of surgeons and nurses felt operating room waste was excessive and should be reduced [8].

The American Medical Association (AMA) House of Delegates, legislative and policy-making arm of the AMA, also has adopted policy in support of both multidosing and post-operative dispensing of medications used intraoperatively [24,25]. While these policies are not law, they are used to advocate for laws reflected in the AMA policies. AMA resolution d-120.929 also states that the AMA will work with federal and state agencies and medical societies to advocate for laws and regulations that authorize discharging patients with partly-used eye drops following a procedure and using multidose eye drops for multiple patients [24].

#### 3.2.4 Hospital and ASC policies

Despite the broad consensus from regulatory and professional bodies, the facility-specific policy remains the most opaque and potent barrier [26]. Research indicates that policies that provide guidance to practitioners on more technical aspects of patient care, such as guidance on prescribing or surgical techniques, are largely unavailable to the public [27,28], and there is not a publicly available database of individual facility policies. Despite this, evidence suggests that surgeons in ASCs are more willing to reuse supplies and medications than those in hospitals [9].

## 4 Discussion

There are few, if any, real barriers to either waste reduction practice. While facility policies remain a potential barrier (Table 3), no federal or state law prohibits these actions. Authorization for dispensing unused medication has been codified in law in several states; of the 38 states with known multidose policies, only two do not authorize. All professional organizations, CMS, and the JC agree that multidosing is acceptable when proper precautions are followed, mitigating infection and malpractice concerns. Further research is needed to determine if facilities prohibit or allow these practices.

**Table 3:** Topics for future legal research on possible barriers to sustainability interventions.

Data Set Topic Suggestions
Supportive state laws
ASC and hospital policies (this will require reaching out to individual facilities to access these)
State laws allowing return and reuse of medications previously dispensed in facility
State standards of care (this will require researching case law)

Therefore, minimizing US ophthalmic drug waste requires action on facility-level policies and perceived regulatory barriers. Professional organizations need alignment with accreditors to counter pervasive, false standards of care, such as the persistent effect of the flawed 2015 CMS checklist. Healthcare facilities must update their internal policies (or make them clear to employees) to reflect the federal and professional consensus. The administrative and IT hurdles of EMR limitations and the financial risk associated with the 340B program must also be addressed with clear, updated guidance to eliminate the administrative incentives for single-use waste.

## 5 Conclusion

Findings of this legal exploration indicate that the primary obstacles to more sustainable cataract surgical practices in the US are *perceived* barriers rooted in misinformation and administrative inertia, rather than explicit legal prohibitions. No federal or state law prohibits multi-dosing or dispensing unused medications. The FDA, CMS, and the Joint Commission have all either explicitly allowed the practice or failed to prohibit it, establishing a clear regulatory floor. Steps should be taken to address perceived barriers and reduce harmful medical waste.

## Sources of financial support

This work was supported by The Patrick and Catherine Weldon Donaghue Medical Research Foundation's Greater Value Portfolio and the National Eye Institute of the National Institutes of Health under Award Number R56EY033779. The content is solely the responsibility of the authors and does not necessarily represent the official views of the National Institutes of Health.

This work was supported in part by an unrestricted grant from Research to Prevent Blindness to the NYU Department of Ophthalmology.

## Authors’ proprietary or financial interests

Dr. Thiel owns Clinically Sustainable Consulting LLC and, through this business, is or has been a paid consultant for the Association for Medical Device Reprocessors (AMDR), Boston Medical Center (BMC), Philips, Augment Surgery (University of Iowa), Becton Dickinson (BD), Veterans Education and Research Association of Northern New England, Inc. (VERANNE), EarthShift Global, Stryker Corporation, CUE Health, Anthesis, Zasti Inc., Sustainable Solutions Corporation, Apiject, Kimberly-Clark Corporation, Sphera, the Institute for Healthcare Improvement (IHI), NYU Stern School of Business, Columbia University’s SHARP program, and the University of California San Francisco (UCSF). She has received honorariums and travel reimbursements for lectures and training given to 3 M, Stryker, Vizient, Columbia University, and the University of Colorado. She is or has been a paid advisor to The Sean N. Parker Center for Allergy and Asthma Research at Stanford University; a paid member of Ambu’s Sustainability Advisory Board; an unpaid member of the Mass General Center for Climate and Health advisory board, the Environmental Sustainability Committee of the Radiological Society of North America (RSNA), and the Working Group on Environmental Sustainability in Medical Imaging Physics (WGESMIP) of the American Association of Physicists in Medicine; and a member of the advisory board for Zabble, Inc. for which she received stock options.

## Declaration of generative AI and AI-assisted technologies in the manuscript preparation process

During the preparation of this work the author(s) used Gemini 2.5 Flash in order to shorten the manuscript to the journal’s required word count. After using this tool/service, the author(s) reviewed and edited the content as needed and take full responsibility for the content of the published article.

## CRediT authorship contribution statement

**Caitlin Davie:** Writing – review & editing, Writing – original draft, Software, Methodology, Investigation, Formal analysis, Data curation. **Lindsay K. Cloud:** Writing – review & editing, Writing – original draft, Visualization, Supervision, Methodology, Investigation, Formal analysis, Data curation, Conceptualization. **Scott Burris:** Writing – review & editing, Supervision, Investigation, Funding acquisition, Conceptualization. **Shaina Shiwdin:** Writing – review & editing, Conceptualization. **Brooke Sherry:** Writing – review & editing, Visualization, Data curation, Conceptualization. **Christina R. Prescott:** Writing – review & editing, Data curation, Conceptualization. **Joel S. Schuman:** Writing – review & editing, Funding acquisition, Conceptualization. **David Palmer:** Writing – review & editing, Validation, Investigation, Formal analysis, Conceptualization. **David Chang:** Writing – review & editing, Methodology, Funding acquisition, Formal analysis, Conceptualization. **Daniel Parra:** Writing – review & editing. **Cordelia Kwon:** Writing – review & editing, Writing – original draft, Visualization, Validation. **Margaret A. Tharp:** Writing – review & editing, Visualization. **Cassandra L. Thiel:** Writing – review & editing, Supervision, Investigation, Funding acquisition, Conceptualization.

## Declaration of competing interest

The authors declare that they have no known competing financial interests or personal relationships that could have appeared to influence the work reported in this paper.
